# SOCIAL INTERACTIONS REDUCE ASSOCIATION BETWEEN LIFE-SPACE AND DAILY HAPPINESS IN OLDER ADULTS WITH AND WITHOUT HIV

**DOI:** 10.1093/geroni/igac059.492

**Published:** 2022-12-20

**Authors:** Lily Kamalyan, Jiue-An Yang, Caitlin Pope, Emily Paolillo, Laura Campbell, Bin Tang, Colin Depp, Raeanne Moore

**Affiliations:** University of California San Diego, San Diego, California, United States; Qualcomm Institute/Calit2, San Diego, California, United States; University of Kentucky, Lexington, Kentucky, United States; University of California San Fransisco, San Francisco, California, United States; University of California San diego, San Diego, California, United States; University of California San Diego, San Diego, California, United States; Department of Psychiatry, University of California San Diego, La Jolla, California, United States; UC San Diego, San Diego, California, United States

## Abstract

**Objective:**

We used global positioning system (GPS) derived indicators and ecological momentary assessment (EMA) to assess real-time relationships between life-space, mood, and social interactions among older adults with and without HIV.

**Methods:**

Participants (People with HIV [PWH] n=54, HIV- n=34) completed smartphone-based EMA surveys assessing mood and social interactions four times/day for 14-days. Participants’ smartphones were GPS enabled. Mixed-effects models analyzed concurrent and lagged associations among life-space and mood.

**Results:**

PWH spent more time at home (79% vs 70%, z=-2.08; p=.04) and reported lower mean happiness (3.2 vs 3.7; z=2.63; p=.007). More daily social interactions were associated with higher ratings of real-time happiness (b=0.12; t=5.61; p< 0.001). Prior day social interactions (b=0.15; t=7.3; p<.0001) and HIV status (b=-0.48; t=-2.56; df= 1026.8; p=0.01) diminished the effect of prior day time spent at home on happiness.

**Conclusions:**

Interventions targeting social interaction may help increase positive mood in isolated older adults.

